# Genomic Sequence Analysis of Granulovirus Isolated from the Tobacco Cutworm, *Spodoptera litura*

**DOI:** 10.1371/journal.pone.0028163

**Published:** 2011-11-23

**Authors:** Yong Wang, Jae Young Choi, Jong Yul Roh, Qin Liu, Xue Ying Tao, Jong Bin Park, Jae Su Kim, Yeon Ho Je

**Affiliations:** 1 Department of Agricultural Biotechnology, College of Agriculture and Life Sciences, Seoul National University, Seoul, Korea; 2 Research Institute for Agriculture and Life Sciences, Seoul National University, Seoul, Korea; Institute of Infectious Disease and Molecular Medicine, South Africa

## Abstract

**Background:**

*Spodoptera litura* is a noctuid moth that is considered an agricultural pest. The larvae feed on a wide range of plants and have been recorded on plants from 40 plant families (mostly dicotyledons). It is a major pest of many crops. To better understand *Spodoptera litura* granulovirus (SpliGV), the nucleotide sequence of the SpliGV DNA genome was determined and analyzed.

**Methodology/Principal Findings:**

The genome of the SpliGV was completely sequenced. The nucleotide sequence of the SpliGV genome was 124,121 bp long with 61.2% A+T content and contained 133 putative open reading frames (ORFs) of 150 or more nucleotides. The 133 putative ORFs covered 86.3% of the genome. Among these, 31 ORFs were conserved in most completely sequenced baculovirus genomes, 38 were granulovirus (GV)-specific, and 64 were present in some nucleopolyhedroviruses (NPVs) and/or GVs. We proved that 9 of the ORFs were SpliGV specific.

**Conclusions/Significance:**

The genome of SpliGV is 124,121 bp in size. One hundred thirty-three ORFs that putatively encode proteins of 50 or more amino acid residues with minimal overlap were determined. No chitinase or cathepsin genes, which are involved in the liquefaction of the infected host, were found in the SpliGV genome, explaining why SpliGV-infected insects do not degrade in a typical manner. The DNA photolyase gene was first found in the genus Granulovirus. When phylogenic relationships were analyzed, the SpliGV was most closely related to *Trichoplusia ni* granulovirus (TnGV) and *Xestia c-nigrum* granulovirus (XecnGV), which belong to the Type I-granuloviruses (Type I-GV).

## Introduction

The family Baculoviridae includes invertebrate-specific viruses with circular, covalently closed, double-stranded DNA genomes ranging in size from 80–180 kb [1]. To date, more than 600 baculoviruses have been described to infect species from the insect orders Lepidoptera, Diptera, and Hymenoptera, and it is likely that baculoviruses represent the largest and most diverse family of DNA viruses [2], [3]. Previously, the family Baculoviridae was subdivided into two genera, *Nucleopolyhedrovirus* (NPV) and *Granulovirus* (GV), mainly based on the morphology of their occlusion bodies (OBs) [4]. Recently, a proposed reclassification has expanded the family to include four genera: the viruses of Lepidoptera are divided into the Alpha- and Beta-baculoviruses, encompassing the NPVs and GVs, respectively, and those infecting Hymenoptera and Diptera are named the Gamma- and Delta-baculoviruses, respectively [5]. While NPVs have OBs with many virions and have been isolated from lepidopteran and non-lepidopteran hosts, the OBs of GVs each contain a single virion and have only been isolated from lepidopteran insects [6]. The lepidopteran-specific NPVs are further classified into two groups, I and II, based on the phylogenetic analysis of their polyhedrin (*polh*) genes [7], [8]. GVs cause three distinct types of pathology in infected hosts [9]: Type I-GVs only infect the fat body, usually resulting in a relatively slow speed of killing; Type II-GVs infect most of the insect host's tissues, resulting in a faster speed of killing; and Type III-GVs infect only the midgut epithelium, resulting in the rapid death of the host. At present, Type III-GVs contain only one member, *Harrisina brillians* granulovirus (HabrGV). Phylogenetic analysis of GV sequences suggests that these different types of GV pathogenesis do not have monophyletic origins [10].

As a novel and steady pesticide, baculoviruses have been used as agents for the biological control of certain insect pest species. Baculoviruses possess several suitable properties, including high efficacy in controlling insect pests and a less negative impact on the environment and non-target species than chemical pesticides [11], [12]. However, their use has been limited due to their slow speed of killing and narrow host specificity. Recent studies have shown that baculoviruses expressing foreign genes, such as a *Bacillus thuringiensis* crystal protein gene and an insect-specific neurotoxin gene have accelerated killing speeds and promise to be effective biological insecticides [13]. To date, GVs have been isolated only from lepidopteran larvae. In particular, GVs infect both agricultural and forest insect pests, making them potentially important as biological insecticides [14].

The tobacco cutworm, *Spodoptera litura*, has totally polyphagous noctuid larvae as well as high reproductive potential and the ability to migrate long distances as adults. The host range of *S*. *litura* covers over 40 families [15]. Among the main crop species attacked by *S*. *litura* in the tropics are *Colocasia esculenta*, cotton, flax, groundnuts, jute, alfalfa, maize, rice, soybeans, tea, tobacco, and vegetables (including eggplants, brassica, capsicum, cucurbit vegetables, wild bean, potatoes, and sweet potatoes). Other hosts include ornamentals, wild plants, weeds, and shade trees (e.g., *Leucaena leucocephala*, the shade tree of cocoa plantations in Indonesia). These factors contribute to the role of *S*. *litura* as a major pest of many agricultural crops throughout its geographical range, and as a result, many insecticide treatments target this pest [16].

Several properties of baculoviruses, such as their relatively slow killing speed, narrow host spectrum, and high production costs, are disadvantageous. To overcome these disadvantages, it is necessary to develop a better understanding of the biology and pathology of baculoviruses. One approach is to conduct extensive research into diverse viruses that possess distinct biological and pathological characteristics. Detailed information about a wide range of isolates will provide a more comprehensive overview of baculoviruses and help to overcome their shortcomings as biological pest control agents. In this study, to add our knowledge of granulovirus molecular genetics, the complete genome of SpliGV was sequenced and analyzed.

## Results and Discussion

### Characteristics of the SpliGV genome sequence

So far, 49 baculovirus genomes have been sequenced (Table 1). Thirty-eight NPV genome sequences have been reported, but to date, complete genome sequences have only been reported for seven GVs [1], [17]–[22], with genome sequences for another four GVs (from *Phthorimaea operculella*, *Agrotis segetum*, *Pseudaletia unipuncta* and *Pieris rapae*) on file in GenBank. A 6× sequence of the SpliGV genome was compiled from all sequence data generated. The size of the final draft sequence was 124,121 nt. The SpliGV genome has an A+T content of 61.2%, which is closest to that of *Pseudaletia unipuncta* GV (60.2%) (PsunGV, GenBank accession no. EU678671). Among GV genomes, that of *Cryptophlebia leucotreta* GV (CrleGV) has the highest A+T content, 67.6%, and that of *Cydia pomonella* granulovirus (CpGV) has the lowest A+T content, 54.8%. Coding sequences represent 86.3% of the genome of SpliGV.

**Table 1:** Characteristics of baculovirus genomes (February 2010).

Virus	No.ORFs	Genomesize (bp)	Accession No.	AT content(%)	Sequenced
*Autographa californica* MNPV	155	133,894	NC_001623	59.3	1994–07–16
*Bombyx mori* NPV	143	128,413	NC_001962	59.6	1996–01–18
*Orgyia pseudotsugata* MNPV	152	131,995	NC_001875	44.9	1997–03–27
*Mamestra configurata* NPV–A	169	155,060	NC_003529	58.3	1997-03–29
*Lymantria dispar* MNPV	166	161,046	NC_001973	42.5	1998–11–03
*Spodoptera exigua* MNPV	139	135,611	NC_002169	56.2	1999-12–29
*Helicoverpa armigera* NPV (G4)	135	131,403	NC_002654	61.0	2001–01-25
*Epiphyas postvittana* NPV	136	118,584	NC_003083	59.3	2001–08–19
*Culex nigripalpus* NPV	109	108,252	NC_003084	49.1	2001–08-22
*Helicoverpa armigera* NPV	134	130,759	NC_003094	61.1	2001–08–31
*Spodoptera litura* NPV	141	139,342	NC_003102	57.2	2001–09–11
*Helicoverpa zea* SNPV	139	130,869	NC_003349	60.9	2002-01–01
*Mamestra configurata* NPV–B	168	158,482	NC_004117	60.0	2002–08–25
*Rachiplusia ou* MNPV	146	131,526	NC_004323	60.9	2002–10–02
*Adoxophyes honmai* NPV	125	113,220	NC_004690	64.4	2003–04–05
*Choristoneura fumiferana* MNPV	145	129,593	NC_004778	49.9	2003–05–06
*Choristoneura fumiferana* DEF NPV	149	131,160	NC_005137	54.2	2003–10–11
*Neodiprion sertifer* NPV	90	86,462	NC_005905	66.2	2004–06–17
*Neodiprion lecontei* NPV	90	81,755	NC_005906	66.7	2004–06–17
*Chrysodeixis chalcites* NPV	151	149,622	NC_007151	60.9	2005–06–29
*Trichoplusia ni* SNPV	144	134,394	NC_007383	61.0	2005–09–07
*Hyphantria cunea* NPV	148	132,959	NC_007767	54.5	2006–02–02
*Agrotis segetum* MNPV	153	147,544	NC_007921	54.3	2006–03–27
*Antheraea pernyi* NPV	147	126,630	NC_008035	46.5	2006–05–16
*Neodiprion abietis* NPV	93	84,264	NC_008252	66.6	2006–07–24
*Clanis bilineata* NPV	129	135,454	NC_008293	62.3	2006–08–24
*Plutella xylostella* MNPV	152	134,417	NC_008349	59.3	2006–09–16
*Leucania separata* NPV	169	168,041	NC_008348	51.4	2006–09–16
*Anticarsia gemmatalis* NPV	152	132,239	NC_008520	55.5	2006–10–21
*Ecotropis obliqua* NPV	126	131,204	NC_008586	62.4	2006–11–21
*Maruca vitrata* MNPV	126	111,953	NC_008725	61.4	2006–12–27
*Spodoptera frugiperda* MNPV	142	131,330	NC_009011	59.6	2007–02–16
*Orgyia leucostigma* NPV	135	156,179	NC_010276	60.1	2008–01–18
*Agrotis ipsilon* MNPV	163	155,122	NC_011345	51.4	2008–10–08
*Helicoverpa armigera* SNPV NNg1	143	132,425	NC_011354	60.9	2008–10–28
*Adoxophyes orana* NPV	121	111,724	NC_011423	65.0	2008–10–28
*Helicoverpa armigera* MNPV	162	154,196	NC_011615	59.9	2008–12–01
*Spodoptera litura* NPV (II)	147	148,634	NC_011616	55.0	2008–12–01
*Xestia c-nigrum* GV	181	178,733	NC_002593	59.3	2000–06–07
*Plutella xylostella* GV	120	100,999	NC_002331	59.3	2000–10–29
*Cydia pomonella* GV	143	123,500	NC_002816	54.8	2001–04–02
*Phthorimaea operculella* GV	130	119,217	NC_004062	64.3	2002–07–01
*Adoxophyes orana* GV	119	99,657	NC_005038	65.5	2003–07–15
*Cryptophlebia leucotreta* GV	129	110,907	NC_005068	67.6	2003–08–13
*Agrotis segetum* GV	132	131,680	NC_005839	62.7	2004–04–09
*Choristoneura occidentalis* GV	116	104,710	NC_008168	67.3	2006–06–19
*Spodoptera litura* GV	133	124,121	NC_009503	61.2	2007–05–30
*Helicoverpa armigera* GV	179	169,794	NC_010240	59.2	2008–01–09
*Pseudaletia unipuncta* GV	183	176,677	EU678671	60.2	2008–10–31
*Pieris rapae* GV	120	108,592	NC_013797	67.0	2010–02–11

**Figure 1:**
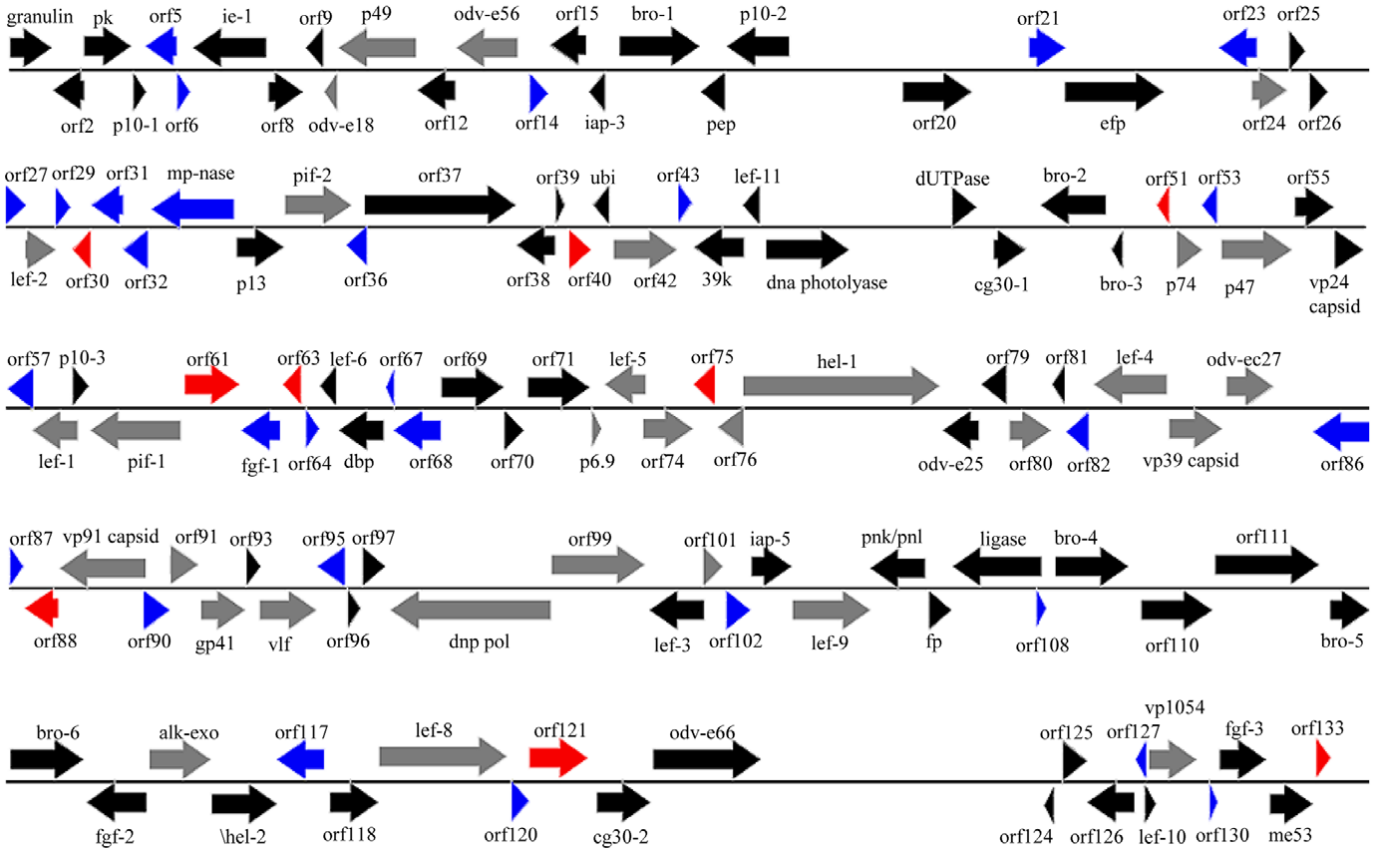
Representation of the SpliGV genome. ORFs and transcriptional direction are indicated by arrows. The ORFs present in most completely sequenced baculovirus genomes are colored gray; GV-specific ORFs are in blue; SpliGV unique ORFs are in red; and ORFs present in some NPVs and/or some GVs are in black.

One hundred thirty-three ORFs of at least 50 codons in length that had minimal overlap with larger ORFs or shared significant sequence identity with previously characterized baculovirus ORFs were identified (Fig. 1). Among these, 31 ORFs were conserved in most completely sequenced baculovirus genomes, 38 were GV-specific, and 64 were present in some NPVs and/or GVs. By convention, the first nucleotide of the methionine start codon of the *granulin* gene was defined as nucleotide 1 of the genome, and the sequence was numbered in the direction of transcription of the *granulin* gene. As with other baculovirus genomes, the ORFs were randomly distributed with 77 ORFs in the *granulin*-sense orientation and 56 in the opposite orientation. Canonical baculovirus early and late gene promoter sequences were associated with 119 ORFs of the SpliGV (Table S1). As we have known, there is little overlap in baculovirus genomic DNAs. Minimal overlaps (less than 16 codons) were observed between 57 adjacent ORFs. More notably, greater levels of overlap were found between Spli22 and Spli23 (62 bp), Spli23 and Spli24 (110 bp), Spli35 and Spli36 (108 bp), Spli58 and Spli59 (110 bp), Spli74 and Spli75 (57 bp), Spli107 and Spli108 (113 bp), and Spli128 and Spli129 (152 bp) (Fig. 1).

**Figure 2:**
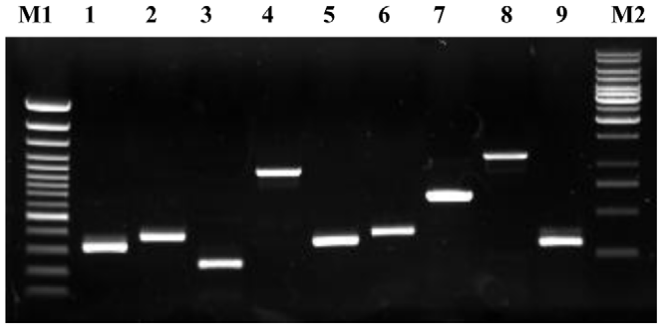
RT-PCR of 9 SpliGV-specific ORFs using mRNAs from *S*. *litura* larvae infected with SpliGV as a template. M1, 100-bp DNA ladder (Fermentas, USA); 1, Spli30; 2, Spli40; 3, Spli51; 4, Spli61; 5, Spli63; 6, Spli75; 7, Spli88; 8, Spli121; 9, Spli133; M2, 1-kb DNA ladder (Fermentas, USA).

BLAST comparisons of the nucleotide sequences and the deduced amino acid sequences of 133 ORFs of SpliGV indicated that 9 ORFs have no similarity to any reported baculovirus genes. We proved that these 9 SpliGV-specific ORFs could be transcribed from the SpliGV genome by RT-PCR (Fig. 2). These 9 ORFs were named Spli30, Spli40, Spli51, Spli61, Spli63, Spli75, Spli88, Spli121, and Spli133. Except for Spli51, the other SpliGV-specific ORFs had some similarities to genes from other microbes.

### Relationships with other baculoviruses

**Figure 3:**
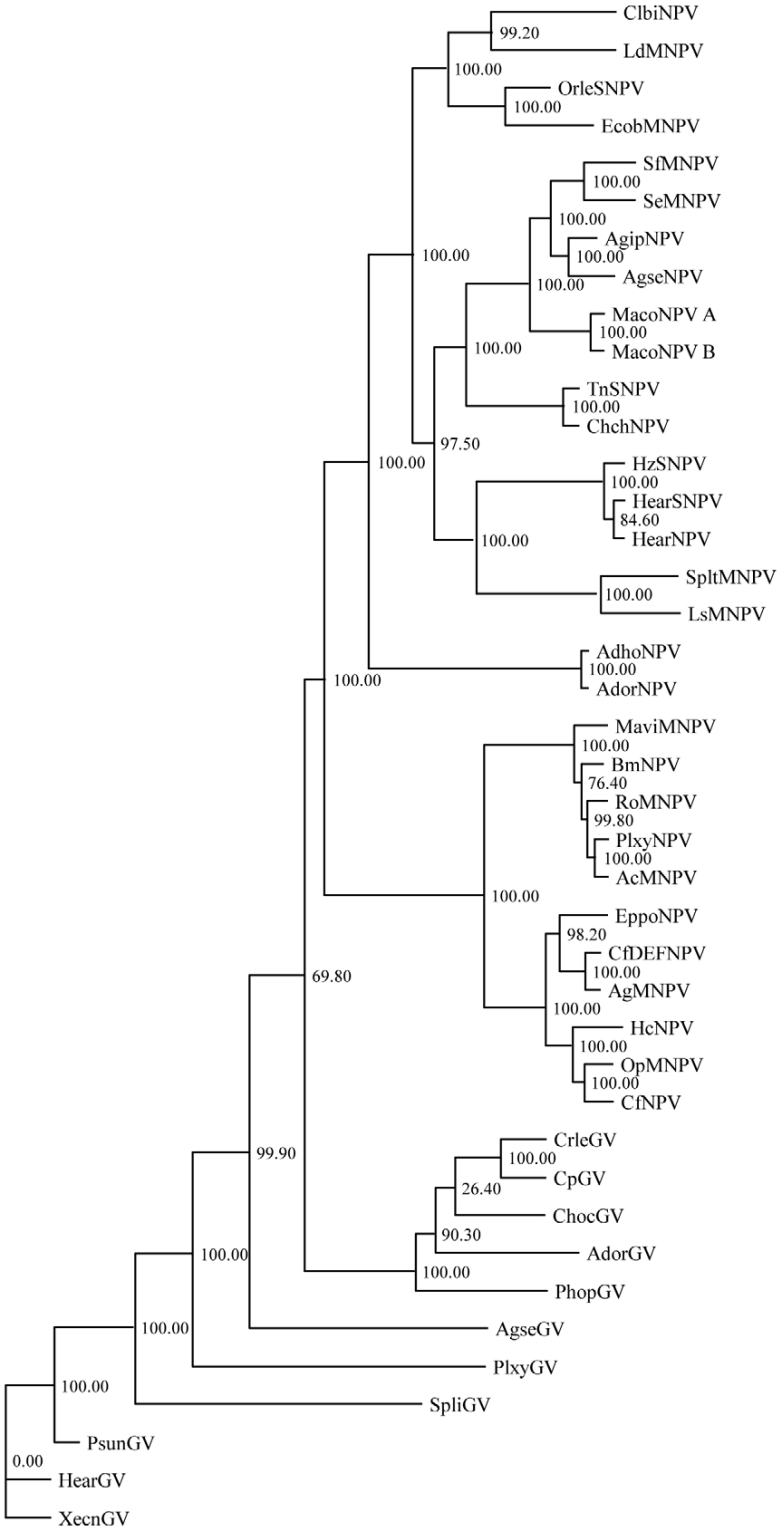
Phylogenic relationship between 41 complete baculovirus genomes based on the nucleotide sequences of 24 genes. The numbers on the branches represent bootstrap values for 1,000 replicates.

Previously, when the nucleotide and the deduced amino acid sequences of the SpliGV granulin gene were aligned with those of granulin and polyhedrin genes from other baculoviruses, SpliGV was most closely related to TnGV and XecnGV, which belong to the Type I-GVs [23]. To further investigate to the relationship between SpliGV and other baculoviruses, phylogenetic trees were inferred from a set of concatenated, aligned, partial amino acid sequences of 24 genes from SpliGV and 40 other completely sequenced lepidopteran baculoviruses (Fig. 3). This result did not confirm a clear relationship between SpliGV and XecnGV, as shown in Figure 3. However, we can place SpliGV and XecnGV in a clade of closely related GVs isolated from Lepidoptera of the family Noctuidae, including AgseGV, *Plutella xylostella* GV (PlxyGV), PsunGV and *Helicoverpa armigera* GV (HearGV). These viruses, along with XecnGV, are considered to be isolates of the same virus species [2].

**Figure 4:**
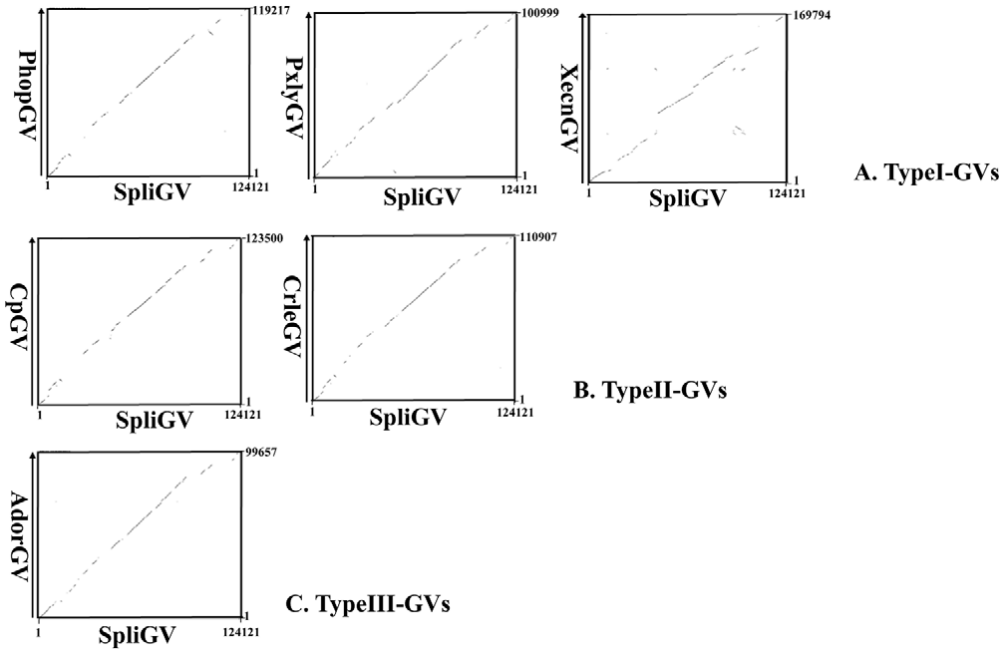
Nucleotide sequence comparison of the SpliGV genome and other GV genomes by matrix dot plot.

Dot plot sequence comparisons revealed a strong degree of co-linearity between the genome of SpliGV and those of other GVs (Fig. 4). SpliGV lacked many of the ORFs found in XecnGV, which is expected given the significantly smaller size of the genome of SpliGV. Comparison of the SpliGV genome with that of *Spodoptera litura* multicapsid NPV (SpltMNPV) revealed that the order of some ORFs was conserved between the two viruses, but the orientation of a large proportion of these ORFs was inverted relative to the polyhedron gene (Fig. 4).

**Figure 5:**
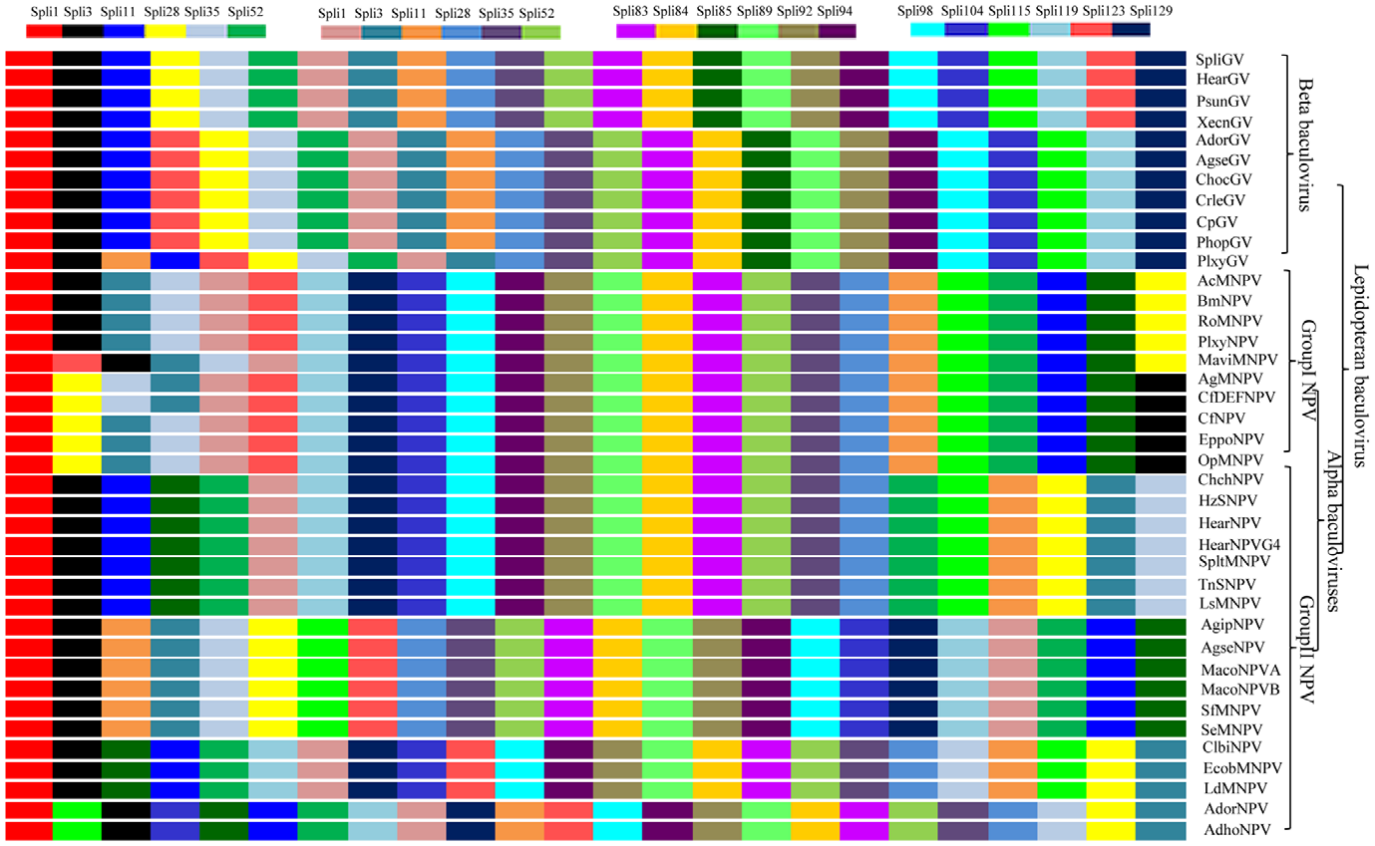
Gene order diagram of 24 genes from 45 complete baculovirus genomes.

Comparative analysis of the gene organization of the SpliGV genome with those of 44 other baculoviruses was carried out using a gene order diagram (GOD) (Fig. 5). The GOD analysis was performed using the 24 genes of the SpliGV genome along with those of 44 other baculoviruses. Homologs of the 24 genes of the SpliGV genome are found in all 40 of the lepidopteran baculovirus genomes. However, some of these homologs have been lost from the three hymenopterous baculovirus genomes and the one dipteran baculovirus genome (Fig. 5). The homologs of Spli73, Spli77, Spli78, Spli83, Spli84, Spli89, Spli92, Spli94, and Spli98 occur in the same order in all 44 lepidopteran baculovirus genomes in either the forward direction or reverse direction relative to the SpliGV genome. In all of the lepidopteran GV genomes, a total of 13 homologs of genes of the SpliGV genome, including Spli55, Spli104, Spli115, and Spli119 as well as the homologs found in all of the lepidopteran baculovirus genomes, are organize in the same order and direction as in the SpliGV genome (Fig. 5). We also found that the order of the SpliGV gene homologs was generally more conserved in the group I NPVs than in the group II NPVs (Fig. 5). The GOD analysis technique may be able to supplement other baculovirus classification methods.

### SpliGV genes involved in DNA replication and transcription

There are 19 *lef* genes in *Autographa californica* multicapsid NPV (AcMNPV) that have been implicated in DNA replication and transcription [24]. Early baculovirus genes are transcribed by the host cell RNA polymerase II, but these genes are often transactivated by genes such as *ie-0*, *ie-1*, *ie-2*, and *pe38* [25]. Among these genes, only *ie-1* is present in the SpliGV genome (Table 2). Both *ie-2* and *pe38* are also absent from all group II NPVs and GVs with the exception of CpGV and PhopGV, which have a *pe38* gene [22]. Six genes have been reported to be essential for baculovirus DNA replication: *lef-1*, *lef-2*, *lef-3*, *dnapol*, *helicase*, and *ie-1* [26]. Homologs of all of these essential genes are present in SpliGV (Table 2). They are moderately well conserved, with the exception of *lef-3* and *ie-1* (Table 2). SpliGV does not have the *lef-7* or *lef-12* typically found in group I NPVs. SpliGV encodes a DNA ligase (Spli107) and a second helicase (Spli116) (Table 2), as do *Lymantria dispar* multicapsid NPV (LdMNPV) and other GVs. In LdMNPV, neither the *helicase-2* nor the *dna ligase* gene stimulates DNA replication in transient assays. As their homologs are involved in DNA repair and recombination, these genes could also be involved in DNA repair [27].

**Table 2:** SpliGV genes grouped according to functional comparison with other baculoviruses.

	Genes present in SpliGV	Genes missing in SpliGV
Transcription	*39 K* (Spli44), *lef-11* (Spli45), p47 (54), lef-6 (Spli 65), *lef-5* (Spli73), *lef-4* (Spli83), *vlf* (Spli94), *lef-9* (Spli104), *lef-8* (Spli119)	*p26* (Ac136), *pe38* (Ac153)
Replication	*ie* (Spli7), *lef-2* (Spli28), *dUTPase* (Spli47), *lef-1* (Spli58), *dbp* (Spli66), *38 k* (Spli74), *hel-1* (Spli77), *dna pol* (Spli98), *lef-3* (Spli100), *pnk/pnl* (Spli105), *dna ligase* (Spli107), *hel-2* (Spli116), *me53* (Spli132)	*ptp* (Ac1), *lef-7* (Ac125), *ie0* (Ac147-0), *nudix* (Ac38), *ie2* (Ac151), *pnk polynucelotide kinase* (Ac33), *rr1* (Cp127), *rr2* (Cp128)
Structural	*granulin* (Spli1), *pk* (Spli3), *p10-1* (Spli4), *odv-e18* (Spli10), *odv-e56* (Spli13), *pep* (Spli18), *p10-2* (Spli19), *efp* (Spli22), *pif-3* (Spli24), *p1 3*(Spli34), *pif-2* (Spli35), *cg30-1* (Spli48), *p74* (Spli52), *vp24capsid* (Spli56), *p10-3* (Spli59), *pif-1* (Spli60), *bv/odv-c42* (Spli71), *p6*.*9* (Spli72), *pif-4* (Spli76), *odv-e25* (Spli78), *p33* (Spli80), *vp39 capsid* (Spli84), *odv-ec27* (Spli85), *vp91capsid* (Spli89), *tlp20* (Spli90), *gp41* (Spli92), *desmop* (Spli99), *fp* (Spli106), *cg30-2* (Spli122), *odv-e66* (Spli123), *vp1054* (Spli129)	*bv/odv-e26* (Ac16), *pkip* (Ac24), *gp64* (Ac128), *vp80*,*vp87* (Ac104), *exon-0* (Ac141)
Auxiliary	*p49* (Spli11), *iap-3* (Spli16), *bro-1* (Spli17), *lef-10* (Spli128), *mp-nase* (Spli33), *ubi* (Spli41), *dna photolyase* (Spli46), *bro-2* (Spli49), *bro-3* (Spli50), *fgf-1* (Spli62), *iap-5* (Spli103), *bro-4* (Spli109), *bro-5* (Spli112), *bro-6* (Spli113), *fgf-2* (Spli114), *alk-exo* (Spli115), *fgf-3* (Spli131)	*p94* (Xecn21), *cathepsin* (Xecn58), *sod* (Xecn68), *chitinase* (Xecn103), *gp37* (Xecn107), *ctl* (Xecn127), *enhancin-1* (Xecn150), *enhancin-2* (Xecn152), *enhancin-3* (Xecn154), *enhancin-4* (Xecn166), *lef-12* (Ac41)
Unknown	Spli2, Spli5, Spli6, Spli8, Spli9, Spli12, Spli14, Spli15, Spli20, Spli21, Spli23, Spli25, Spli26, Spli27, Spli29, Spli31, Spli32, Spli36, Spli37, Spli38, Spli39, Spli42, Spli43, Spli53, Spli55, Spli57, Spli64, Spli67, Spli68, Spli69, Spli70, Spli79, Spli81, Spli82, Spli86, Spli87, Spli91, Spli93, Spli95, Spli96, Spli97, Spli101, Spli102, Spli108, Spli110, Spli111, Spli117, Spli118, Spli120, Spli124, Spli125, Spli126, Spli127, Spli130	
SpliGV unique	Spli30, Spli40, Spli51, Spli61, Spli63, Spli75, Spli88, Spli121, Spli133	

SpliGV lacks genes for enzymatic functions in nucleotide metabolism, such as the large (*rr1*) and small (*rr2*) subunits of ribonucleotide reductase, but it does have the deoxyuridyltriphosphate (*dUTPase*) gene (Table 2). These enzymes are found in several baculoviruses and are involved in nucleotide metabolism. They catalyze the reduction of host cell rNTPs to dNTPs [1].

Many genes required for late gene transcription have been described, including *lef-4*, *lef -6*, *lef -8*, *lef -9*, *lef -10*, *lef -11*, *39 k*, *p47*, and *vlf-1* [28]. All of these genes are found in SpliGV (Table 2). Generally, these genes are more conserved than the early transcription activators [29]. The *lef-6* genes of GVs are smaller than the *lef-6* genes of NPVs (86–102 amino acids vs. 138–187 amino acids).

### SpliGV structural genes

The most conserved baculovirus structural protein is polyhedrin/granulin (93% maximal amino acid identity), the major component of OBs [30]. SpliGV lacked homologs of two structural genes, the *p80/87-capsid* gene and ORF 1629 (*p78/73*). The *p80/87-capsid* gene is also absent from the other sequenced GVs. A putative ORF 1629 (Xecn2) has been identified in XecnGV (Xecn2), although it is less than half the size of the NPV ORFs and has similarity concentrated only around a conserved proline-rich region [20]. Except for HearGV, all of the GVs have a Xcen2 homolog with similarity in the first 65 aa of the amino-terminal region of the protein but show no similarity to ORF 1629 and do not contain a proline-rich region. *Pep* is found on the surface of the OBs and is important for the formation of polyhedra, as it stabilizes them and prevents them from fusing [31]. SpliGV does have a *pep* (Spli18) that shares 55% amino acid identity with its homolog from XecnGV, Xecn18 (Table 2).

Occlusion-derived viruses (ODVs) contain more than 10 different envelope proteins. Five of these, denoted *pif-1*, *pif-2*, *pif-3*, *pif-4*, and *p74*, have been identified as essential for *per os* infection of insect larvae [32]–[36]. All five proteins are highly conserved in Baculoviridae and are encoded by the so-called core genes [32], [37]–[39]. These PIF proteins function in the early stage of virus infection, and deletion of any of these *pif* genes leads to a block in infection prior to viral gene expression in midgut epithelial cells [33], [35], [40]. All five genes involved in *per os* infectivity were present in SpliGV (Table 2). In the SpliGV genome, *p74* encodes a small, truncated protein (144 aa) containing the conserved C-terminal region of the XcenGV *p74* (Xcen77) gene (43% identity over 140 aa), but the *p74* of SpliGV is substantially shorter than that of XcenGV (144 aa vs. 710 aa).

In NPV-infected cells, P10 forms fibrillar structures in the nucleus and cytoplasm [21]. This protein is implicated in OB morphogenesis and disintegration of the nuclear matrix, resulting in the dissemination of OBs [41]. P10 proteins from different baculoviruses are characterized by the size differences in their shared domains, and their sequences are generally poorly conserved [17]. Three XcenGV ORFs (Xcen5, Xcen19, and Xcen83) present similarities to *p10*. Homologs of these three ORFs are present in PlxyGV (Plxy2, Plxy21, and Plxy50), and Hashimoto *et al*. [19] suggested that they are all *p10* homologs. SpliGV contained homologs of the three putative *p10* genes of XcenGV, which are Spli4, Spli19, and Spli59 (Table 2). They showed maximal similarity to Xcen5, Xcen19, and Xcen83, respectively. Whereas Spli4 (*p10*–*1*) and Spli19 (*p10*–*2*) have a proline-rich domain and a heptad sequence, Spli59 (*p10*–*3*) has only a heptad sequence significantly smaller than that of Xcen83 (97 vs. 182 amino acids). The close association of P10 and the polyhedron envelope has been well documented in many NPVs, and it has been known that the presence of P10 is essential for the formation of the polyhedron envelope [17]. In some GVs, the functional association of PEP and P10 might have been conserved in a single protein [1]. Further work must be done to fully understand the role of the different *p10* homologs in SpliGV.

### SpliGV auxiliary genes

Auxiliary genes are not essential for viral replication, but they do provide some selective advantages [42]. SpliGV does not contain either a *chitinase* or a *cathepsin* gene. It appears that baculoviruses encode these enzymes to aid breakdown of insect tissues at the end of infection to release OBs into the environment and thereby aid their horizontal spread. SpliGV-infected larvae do not lyse at the end of infection. The cadavers of *S*. *litura* larvae infected by SpliGV appeared smaller than normal larvae, as if they had lost much water, and they were very soft.

Enhancin is a metalloproteinase that disrupts the insect peritrophic membrane, facilitating the initiation of infection [43], [44]. These genes were first found in GV OBs, which can enhance the infection of some NPVs. Also referred to as viral enhancing or synergistic factors, enhancins were first identified and isolated by Tanada and colleagues [45]. Enhancin genes have been found in several GVs, including HearGV [46], PsunGV [46], TnGV [47], XecnGV [20], AgseGV (GenBank: AY522332), and ChfuGV (GenBank: AAG33872). The first GV genome to be completely sequenced, that of XecnGV, was found to have four different *enhancin* genes [20]. In contrast, no *enhancin* homolog is present in SpliGV.

Superoxide dismutase (*sod*) is a well-conserved gene of baculoviruses. This gene is presumably involved in the removal of free radicals but is non-essential, and its role in the virus life cycle is not known [48]. Of all of the GVs that have been sequenced to date, SpliGV is not the only one to lack *sod*; *sod* is also not found in a few NPV genomes.

Ubiquitin is the most conserved auxiliary gene and is present in all sequenced baculovirus genomes. The main function of cellular ubiquitin is to signal protein degradation [49]. Viral ubiquitin is nonessential, and its role is unclear [50]. SpliGV contains a homolog of ubiquitin, Spli41 (Table 2).

Another SpliGV ORF of interest is Spli46, a homolog of the DNA *photolyase* gene (Table 2). DNA *photolyase* genes encode photoactive enzymes that are involved in the repair of UV-damaged DNA [51]. To date, in the sequenced baculoviruses other than SpliGV, *photolyase* genes are found only in ChchNPV and *Trichoplusia ni* NPV (TnNPV).

### Inhibitors of apoptosis (IAP)

Apoptosis represents an important virus-host interaction process that probably influences viral pathogenesis. As an antiviral response in multicellular organisms, apoptosis can limit viruses in the suicide cells, thereby reducing the yield of progeny viruses, which results in abortive infection [52], [53]. Although many viruses, including baculoviruses, can trigger apoptosis in infected cells, they can synthesize proteins that prevent apoptosis [54]–[57]. Baculoviruses possess two families of genes that suppress apoptosis, the *p35/p49* family and the inhibitor of apoptosis (IAP) family. P35 was the first antiapoptotic baculovirus protein discovered, and it has only been indentified in AcMNPV, *Bombyx mori* NPV (BmNPV), SpliMNPV, and *Maruca vitrata* MNPV (MaviMNPV) [58]–[60]. A larger *p35* homolog, *p49*, has been found in some baculoviruses [57], [60]. P49 has a similar three-dimensional structure and the same mode of action as P35. However, P49 is able to inhibit initiator caspases that P35 is unable to inhibit [57], [61], [62]. All baculoviruses have been found to contain IAP homologs. The IAP-3 protein of CpGV was the first member of the baculovirus IAP family to be indentified [56]. IAP homologs generally contain two baculovirus IAP repeats (BIR), which are associated with binding to apoptosis-inducing proteins, and a C-terminal zinc finger-like (RING) *Cys*/*His* motif [54], [56], [63]. According to amino acid sequence similarity, baculovirus IAPs can be divided into five types, named *iap*-1 through *iap*-5 [21]. There are two IAP genes, *iap*-3 (Spli16) and *iap*-5 (Spli103), and one *p49* (Spli11) gene in the genome of SpliGV (Table 2).

### Baculovirus repeated ORFs (*bro* genes)

One to sixteen copies of *bro* are present in all lepidopteran and dipteran NPVs sequenced to date and in some of GVs. They comprise a highly repetitive and conserved gene family that is widespread among insect DNA viruses [64]. Gene expression, nucleic acid binding activity, nucleosome association, protein localization and protein trafficking have been characterized for some NPV *bro* genes and proteins, but the functions of *bro* gene products in the baculovirus life cycle are still unclear [64]–[68]. SpliGV has 6 *bro* genes, named *bro*-1 to *bro*-6 based on their order in the genome, including homologs of XecnGV *bro*-a (Xecn60), *bro*-b (Xecn76), *bro*-e (Xecn130), and *bro*-f (Xecn131) (Table 2). In SpliGV, there are two adjacent pairs of *bro* ORFs (*bro*-2 and *bro*-3; *bro*-5 and *bro*-6). Interestingly, the *bro*-1, *bro*-4, and *bro*-6 genes are all homologs of Xecn60 (Table 2). The *bro*-3 gene of SpliGV encodes a small, truncated protein (67 aa) containing the conserved N-terminal region of the Xecn76 homolog.

## Materials and Methods

### Viral DNA extraction

The granules produced in larval cadavers were purified by a standard method [69]. To extract virus DNA, the purified granules were resuspended in 0.1 M sodium carbonate solution [0.1 M Na_2_CO_3_, 0.17 M NaCl, 0.01 M EDTA (pH 10.9)] and incubated at 37°C overnight with 0.5 mg/ml proteinase K (Sigma) and 1% SDS. A further extraction with phenol and chloroform:isoamyl alcohol (24∶1) was performed, and the DNA was ethanol precipitated. The DNA was resuspended in TE buffer [10 mM Tris-HCl, pH 8; 1 mM EDTA].

### Sequencing and sequence analysis of SpliGV genomic DNA

The complete nucleotide sequence of SpliGV genomic DNA was determined using a shotgun strategy on an ABI Model 3700 sequencer (PE-Applied Biosystems, USA). The SpliGV DNA sequence was determined at least six times, and additional assessments were carried out for ambiguous sequences using gene-specific primers. Putative coding regions of the SpliGV genome were predicted using FGENESV0 (http://www.softberry.com/berry.phtml) [70] and the NCBI ORF finder (http://www.ncbi.nlm.nih.gov/gorf/gorf.html) by locating translation start and stop codons of ORFs of 50 or more amino acids. Dot plot sequence comparisons were generated with Advanced PipMaker (http://pipmaker.bx.psu.edu/cgi-bin/pipmaker?advanced). Predicted amino acid sequence identities were obtained from the results of protein database searches using the standard protein-protein BLAST algorithm (http://blast.ncbi.nlm.nih.gov/Blast.cgi). The genomic DNA sequence was deposited in GenBank under the accession number NC990503.

### Phylogenetic analysis

For phylogenetic analysis, 24 genes from 30 NPVs and 10 GVs, which all have homologs in the SpliGV genome, were obtained from GenBank. Phylogenetic analysis was carried out using the maximum-parsimony (MP) method [71] incorporated in the parsimony program PAUP (Phylogenetic Analysis Using Parsimony and Other Methods) version 4.0 b10 program [72]. The reliability of the trees was tested with bootstrap re-sampling using 1,000 replicates.

### Total RNA extraction and RT-PCR

Total RNA was isolated from infected *S*. *litura* larvae with TRIZOL^®^ Reagent (Invitrogen, USA) according to the manufacturer's instructions. Reverse transcription polymerase chain reaction (RT-PCR) was performed using AccuPower^®^ RT/PCR PreMix (Bioneer, Korea) with SpliGV unique gene primer sets (Table S2). One microgram of total RNA and 20 pmol reverse primers were mixed, incubated at 70°C for 5 min and placed on ice. The incubated mixtures and 20 pmol forward primers were transferred to an AccuPower^®^ RT/PCR PreMix tube, and the reaction volumes were brought up to 20 microliters with DEPC-DW. cDNA synthesis reactions and DNA PCR reactions were performed under the following temperature cycles: one cycle at 42°C for 60 min; 94°C for 5 min; one cycle at 94°C for 30 sec, 50°C for 30 sec, and 72°C for 90 sec; 33 cycles at 94°C for 30 sec, 55°C for 30 sec, and 72°C for 90 sec; and 1 cycle at 94°C for 30 sec, 55°C for 30 sec, and 72°C for 7 min.

## Supporting Information

Table S1 **Predicted SpliGV ORFs by BLAST Search.**
(DOC)

Table S2 **Primers used to confirm new SpliGV genes.**
(DOC)
