# Supplementary material for: Genomic Sequence Analysis of Granulovirus Isolated from the Tobacco Cutworm, Spodoptera litura
Source: PLoS One. 2011 Nov 23;6(11):e28163. doi: 10.1371/journal.pone.0028163 (PMC3223241; doi:10.1371/journal.pone.0028163)
Supplement: Table S1 — Predicted SpliGV ORFs by BLAST Search. (DOC) [file pone.0028163.s001.doc]

Table S1. Predicted SpliGV ORFs by BLAST Search

| ORFs | Name | Position | Promoter  motifs | Length  (aa) | Best-matched baculovirus ORF (Protein ID) (% aa identity) |
| --- | --- | --- | --- | --- | --- |
| 1 | *granulin* | 1→750 | L | 249 | *Xestia c-nigrum* granulovirus *granulin* (AAF05115) (93% over 249 aa) |
| 2 | ORF 2 | 747←1316 | L | 189 | *X. c-nigrum* granulovirus ORF 2 (AAF05116) (46% over 65 aa) |
| 3 | *pk* | 1297→2145 | E/L | 282 | *Agrotis segetum g*ranulovirus ORF 3 (AAS82735) (59% over 281 aa) |
| 4 | *p10-1* | 2161→2397 | L | 78 | *X. c-nigrum* granulovirus ORF 5 (AAF05119) (59% over 67 aa) |
| 5 | ORF 5 | 2369←2938 | L | 189 | *Phthorimaea operculella* granulovirus hypothetical protein (AAM70202) (49% over 187 aa) |
| 6 | ORF 6 | 2937→3170 | L | 77 | *A. segetum* granulovirus ORF 7 (AAS82713) (43% over 74 aa) |
| 7 | *ie-1* | 3210←4508 |  | 432 | *X. c-nigrum* granulovirus ORF 9 (AAF05123) (41% over 363 aa) |
| 8 | ORF 8 | 4539→5156 |  | 205 | *X. c-nigrum* granulovirus ORF 10 (AAF05124) (38% over 207 aa) |
| 9 | ORF 9 | 5202←5501 | L | 99 | *X. c-nigrum* granulovirus ORF 11 (AAF05125) (66% over 99 aa) |
| 10 | *odv-e18* | 5512←5754 | L | 80 | *X. c-nigrum granulovirus* ORF 12 (AAF05126) (74% over 83 aa) |
| 11 | *p49* | 5760←7130 | E/L | 456 | *X. c-nigrum granulovirus* ORF 13 (AAF05127) (48% over 458 aa) |
| 12 | ORF 12 | 7145←7828 |  | 227 | *X. c-nigrum granulovirus* ORF14 (AAF05128) (29% over 231 aa) |
| 13 | *odv-e56* | 7828←8925 | E/L | 365 | *Choristoneura occidentalis* granulovirus *odv-e56* (ABC61148) (54% over 348 aa) |
| 14 | ORF 14 | 9122→9454 | E/L/E | 110 | *Plutella. xylostella* granulovirus ORF 18 (AAG27316) (44% over 34 aa) |
| 15 | ORF 15 | 9473←10120 | L | 215 | *Cryptophlebia leucotreta* granulovirus unknown (AAQ21617) (56% over 208 aa) |
| 16 | *iap-3* | 10155←10448 |  | 97 | *Trichoplusia ni granulovirus inhibitor of apoptosis protein* (AAF19819) (59% over 94 aa) |
| 17 | *bro-1* | 10702→12117 | L | 471 | *Chrysodeixis chalcites* nucleopolyhedrovirus *bro-b* (AAY84000) (45% over 466 aa) |
| 18 | *pep* | 12119←12553 | L | 144 | *X. c-nigrum* granulovirus ORF 18 (AAF05132) (55% over 136 aa) |
| 19 | *p10-2* | 12574←13686 | L | 370 | *X. c-nigrum* granulovirus ORF 19 (AAF05133) (57% over 375 aa) |
| 20 | ORF 20 | 15666→16898 | L | 410 | *X. c-nigrum* granulovirus ORF 25 (AAF05139) (26% over 456 aa) |
| 21 | ORF 21 | 17881→18534 | E | 217 | *X. c-nigrum g*ranulovirus ORF 26 (AAF05140) (26% over 190 aa) |
| 22 | *efp* | 18512→20269 | L | 585 | *A. segetum* granulovirus ORF 25 (AAS82713) (45% over 542 aa) |
| 23 | ORF 23 | 21208←21897 | L | 229 | *X. c-nigrum* granulovirus ORF 29 (AAF05413) (41% over 155 aa) |
| 24 | *pif-3* | 21788→22435 | E/L | 215 | *P. xylostella* granulovirus ORF 29 (**AAG27327**) (47% over 186 aa) |
| 25 | ORF 25 | 22451→22738 | L | 95 | *P. xylostella* nucleopolyhedrovirus 11k-like peptide (**ABE68533**) (30% over 90 aa) |

Table S1. Continued

| ORFs | Name | Position | Promoter  motifs | Length  (aa) | Best-matched baculovirus ORF (Protein ID) (% aa identity) |
| --- | --- | --- | --- | --- | --- |
| 26 | ORF 26 | 22807→23139 | L | 110 | *Spodoptera. litura* nucleopolyhedrovirus unknown (AAL01708) (35% over 92 aa) |
| 27 | ORF 27 | 23994→24344 | L | 116 | *X. c-nigrum* granulovirus ORF 34 (AAF05148) (54% over 103 aa) |
| 28 | *lef-2* | 24322→24846 | E,E/L | 174 | *A. segetum* granulovirus ORF 35 (AAS82703) (50% over 171 aa) |
| 29 | ORF 29 | 24839→25084 | L | 81 | *A. segetum granulovirus* ORF 36 (AAS82702) (50% in 76 aa) |
| 30 | ORF 30 | 25115←25426 | E/L | 103 | *LOW-QUALITY PROTEIN: phage integrase (EFL27449) (35% over 64 aa)* |
| 31 | ORF 31 | 25423←25980 | L | 185 | *A. segetum* granulovirus ORF 40 (AAS826989) (31% over 131 aa) |
| 32 | ORF 32 | 25958←26380 | L | 140 | *X. c-nigrum* granulovirus ORF 39 (AAF05153) (27% over 124 aa) |
| 33 | *mp-nase* | 26435←27829 | E | 464 | *X. c-nigrum g*ranulovirus ORF 40 (AAF05154) (37% over 427 aa) |
| 34 | *p13* | 27868→28671 | E | 267 | *X. c-nigrum* granulovirus ORF 43 (AAF05157) (50% over 266 aa) |
| 35 | *pif-2* | 28677→29813 | E/L | 378 | *X. c-nigrum* granulovirus ORF 45 (AAF05159) (58% over 376 aa) |
| 36 | ORF 36 | 29706←30065 |  | 119 | *X. c-nigrum* granulovirus ORF 46 (AAF05160) (30% over 60 aa) |
| 37 | ORF 37 | 30019→32565 | L | 848 | *X. c-nigrum* granulovirus ORF 47 (AAF05161) (34% over 207 aa) |
| 38 | ORF 38 | 32555←33217 | E/L | 220 | *P. xylostella* granulovirus ORF 40 (AAG27388) (84% over 182 aa) |
| 39 | ORF 39 | 33224→33385 | E | 53 | *P. xylostella* granulovirus ORF 41 (AAG27339) (51% in 52 aa) |
| 40 | ORF 40 | 33449→33820 | E | 123 | *oxidoreductase, short-chain dehydrogenase/ reductase family (XP_001823260.2) (29% over 120 aa)* |
| 41 | *ubi* | 33846←34127 | L | 93 | *A. segetum* granulovirus *Ubiquitin* (AAS82691) (85% over 90 aa) |
| 42 | ORF 42 | 34198→35274 | E/L | 358 | *A. segetum* granulovirus ORF 48 (AAS82690) (55% over 361 aa) |
| 43 | ORF 43 | 35286→35522 | L | 78 | *X. c-nigrum* granulovirus ORF 54 (AAF05168) (47% over 72 aa) |
| 44 | *39k* | 35534←36391 | L | 285 | *X. c-nigrum* granulovirus ORF 55 (AAF05169) (62% over 241 aa) |
| 45 | *lef-11* | 36354←36650 | E/L | 98 | *X. c-nigrum* granulovirus ORF 56 (AAF05170) (51% over 89 aa) |
| 46 | *dna photolyase* | 36756→38156 | L | 466 | *C. chalcites* nucleopolyhedrovirus *dna photolyase 2* (AAY84003) (52% over 456 aa) |
| 47 | *dUTPase* | 39873→40295 |  | 140 | *A. segetum* granulovirus ORF 17 (AAS82721) (47% over 137 aa) |
| 48 | *cg30-1* | 40570→41124 | E | 184 | *S. frugiperda* nucleopolyhedrovirus cg30 (ABM45788) (37% over 62 aa) |
| 49 | *bro-2* | 41353←42459 | E | 368 | *X. c-nigrum* granulovirus ORF 131 (AAF05245) (41% over 365 aa) |
| 50 | *bro-3* | 42543←42746 | E | 67 | *S. litura* nucleopolyhedrovirus *hypothetical protein* (AAL01807) (84% over 63 aa) |

Table S1. Continued

| ORFs | Name | Position | Promoter  motifs | Length  (aa) | Best-matched baculovirus ORF (Protein ID) (% aa identity) |
| --- | --- | --- | --- | --- | --- |
| 51 | ORF 51 | 43299←43529 | E | 76 | no significant match |
| 52 | *p74* | 43646→44080 |  | 144 | *X. c-nigrum* granulovirus ORF 77 (AAF05191) (43% over 140 aa) |
| 53 | ORF 53 | 44061←44324 | L | 87 | *C. pomonella* granulovirus ORF 62 (AAK70722) (54% over 59 aa) |
| 54 | *p47* | 44392→45585 | E/L/L | 397 | *X. c-nigrum* granulovirus ORF 78 (AAF05192) (62% over 394 aa) |
| 55 | *ORF 55* | 45621→46289 | L | 222 | *X. c-nigrum* granulovirus ORF 79 (AAF05193) (76% over 223 aa*)* |
| 56 | *vp24 capsid* | 46295→46783 | E/L | 162 | *X. c-nigrum* granulovirus ORF 80 (AAF05194) (60% over 151 aa) |
| 57 | ORF 57 | 46794←47255 | E/E/L | 153 | *P. xylostella* granulovirus ORF 54 (AAG27352) (31% over 135 aa) |
| 58 | *lef-1* | 47227←48060 | L | 277 | *X. c-nigrum* granulovirus ORF 82 (AAF05196) (57% over 236 aa) |
| 59 | *p10-3* | 47951→48244 | E | 97 | *Lymantria* dispar nucleopolyhedrovirus *p10* (AAC70226) (34% over 84 aa) |
| 60 | *pif-1* | 48259→49875 | L | 538 | *P. xylostella* granulovirus ORF 7 (AAG27305) (50% over 502 aa) |
| 61 | ORF 61 | 49945→50916 | E/L | 323 | Endonuclease/exonuclease/phosphatase family protein (XP_002382582.2) (28% over 133 aa) |
| 62 | *fgf-1* | 50932←51630 | L | 232 | *A. segetum* granulovirus ORF 66 (AAS82672) (37% over 232 aa) |
| 63 | ORF 63 | 51675←52007 | L | 111 | hypothetical protein FG06345.1 (XP_386521.1) (46% over 42 aa) |
| 64 | ORF 64 | 52078→52323 | E/L | 81 | *Adoxophyes orana* granulovirus ORF 66 (AAP85703) (38% over 83 aa) |
| 65 | *lef-6* | 52320←52619 | L | 99 | *X. c-nigrum* granulovirus ORF 88 (AAF05202) (40% over 92 aa) |
| 66 | *dbp* | 52653←53465 | L | 270 | *P. xylostella* granulovirus ORF 61 (AAG27359) (35% over 264 aa) |
| 67 | ORF 67 | 53500←53652 | L | 50 | *P. xylostella* granulovirus ORF 62 (AAG27360) (50% over 34 aa) |
| 68 | ORF 68 | 53624←54475 | L | 283 | *X. c-nigrum* granulovirus ORF 90 (AAF05204) (27% over 101 aa) |
| 69 | ORF 69 | 54474→55595 | E/L | 373 | *X. c-nigrum* granulovirus ORF 91 (AAF05205) (64% over 370 aa) |
| 70 | ORF 70 | 55596→55934 | E | 112 | *X. c-nigrum* granulovirus ORF 92 (AAF05206) (52% over 117 aa) |
| 71 | *odv/odv-c42* | 56004→57113 | E/L | 369 | *X. c-nigrum* granulovirus ORF 93 (AAF05207) (60% over 375 aa) |
| 72 | *p6.9* | 57132→57314 | L | 61 | *X. c-nigrum* granulovirus ORF 94 (AAF05208) (59% over 63 aa) |
| 73 | *lef-5* | 57358←58101 | L | 247 | *P. xylostella* granulovirus ORF 69 peptide (AAG27367) (57% over 246 aa) |
| 74 | *38k* | 58045→58944 | L | 299 | *X. c-nigrum* granulovirus ORF 96 (AAF05210) (52% over 288 aa) |
| 75 | ORF 75 | 58937←59314 | E | 125 | secreted protein containing DUF1028 (YP_863458.1) (26% over 130 aa) |

Table S1. Continued

| ORFs | Name | Position | Promoter  motifs | Length  (aa) | Best-matched baculovirus ORF (Protein ID) (% aa identity) |
| --- | --- | --- | --- | --- | --- |
| 76 | *pif-4* | 59355←59834 | E/L | 159 | *X. c-nigrum* granulovirus ORF 97 (AAF05211) (58% over 140 aa) |
| 77 | *hel-1* | 59818→63306 | L | 1162 | *X. c-nigrum* granulovirus ORF 98 (AAf05212) (64% over 1165 aa) |
| 78 | *odv-e25* | 63337←63990 | L | 217 | *C. pomonella* granulovirus *odv-e25* (AAK70751) (70% over 217 aa) |
| 79 | ORF 79 | 64016←64483 | L | 155 | *P. xylostella* granulovirus ORF 75 (AAG27373) (50% over 155 aa) |
| 80 | *p33* | 64520→65275 | L | 251 | *X. c-nigrum* granulovirus ORF 101 (AAF05215) (53% over 250 aa) |
| 81 | ORF 81 | 65272←65508 | L | 78 | *A. segetum* granulovirus ORF 84 (AAS82654) (61% over 63 aa) |
| 82 | ORF 82 | 65528←65926 | L | 132 | *P. xylostella* granulovirus ORF 77 (AAG27375) (33% over 100 aa) |
| 83 | *lef-4* | 66007←67323 | L | 438 | *X. c-nigrum* granulovirus ORF 110 (AAF05224) (51% over 445 aa) |
| 84 | *vp39 capsid* | 67348→68301 | L | 317 | *X. c-nigrum* granulovirus ORF111 (AAF05225) (56% over 327 aa) |
| 85 | *odv-ec27* | 68362→69216 | L | 284 | *X. c-nigrum* granulovirus ORF 112 (AAF05226) (62% over 288 aa) |
| 86 | ORF 86 | 69881←70903 | L | 340 | *A. segetum* granulovirus ORF 88 (AAS82650) (32% over 361 aa) |
| 87 | ORF 87 | 70945→71223 | L | 92 | *A. segetum* granulovirus ORF 90 (AAS82648) (61% over 52 aa) |
| 88 | ORF 88 | 71224←71880 | E | 218 | Hypothetical protein LELG_01697 (XP_001526869.1) (23% over 224 aa) |
| 89 | *vp91 capsid* | 71889←73562 | L | 557 | *P. xylostella* granulovirus ORF 84 (AAG27382) (37% over 531 aa) |
| 90 | *tlp20* | 73519→74040 | E/L | 173 | *A. segetum* granulovirus ORF 93 (AAS82645) (33% over 175 aa) |
| 91 | ORF 91 | 74024→74593 | E/L | 189 | *X. c-nigrum* granulovirus ORF 120 (AAF05234) (62% over 186 aa) |
| 92 | *gp41* | 74629→75486 | E/L | 285 | *X. c-nigrum* granulovirus ORF 121 (AAF05235 ) (60% over 290 aa) |
| 93 | ORF 93 | 75491→75772 | L | 93 | *X. c-nigrum* granulovirus ORF 122 (AAF05236 ) (39% over 83 aa) |
| 94 | *vlf* | 75744→76862 | L | 372 | *X. c-nigrum* granulovirus ORF 123 (AAF05237) (66% over 355 aa) |
| 95 | ORF 95 | 76843←77388 | E | 181 | *A. segetum* granulovirus ORF 98 (AAS82640) (40% over 175 aa) |
| 96 | ORF 96 | 77446→77703 | E/L | 85 | *X. c-nigrum* granulovirus ORF 125 (AAF05239) (77% over 85 aa) |
| 97 | ORF 97 | 77727→78173 | L | 148 | *X. c-nigrum* granulovirus ORF 126 (AAF05240) (43% over 147 aa) |
| 98 | *dna pol* | 78252←81359 | E | 1035 | *X. c-nigrum* granulovirus ORF 132 (AAF05246) (62% over 1006 aa) |
| 99 | *Desmop* | 81361→83172 | E | 603 | *X. c-nigrum* granulovirus ORF 133 (AAF05247) (30% over 702 aa) |
| 100 | *lef-3* | 83229←84305 |  | 358 | *X. c-nigrum* granulovirus ORF 134 (AAF05248) (33% over 364 aa) |

Table S1. Continued

| ORFs | Name | Position | Promoter  motifs | Length  (aa) | Best-matched baculovirus ORF (Protein ID) (% aa identity) |
| --- | --- | --- | --- | --- | --- |
| 101 | ORF 101 | 84280→84669 | L | 129 | *A. segetum* granulovirus ORF 104 (AAS82634) (52% over 123 aa) |
| 102 | ORF 102 | 84713→85192 | E | 159 | *A. orana* granulovirus ORF 98 (AAP85735) (30% over 163 aa) |
| 103 | *iap-5* | 85198→85989 |  | 263 | *P. xylostella* granulovirus ORF 98 (AAG27396) (39% over 268 aa) |
| 104 | *lef-9* | 85997→87508 |  | 503 | *X. c-nigrum* granulovirus ORF 139 (AAF05253) (71% over 490 aa) |
| 105 | *pnk / pnl* | 87476←88558 | E | 360 | *A. gemmatalis* nucleopolyhedrovirus *pnk/pnl* (ABI13886) (41% over 372 aa) |
| 106 | *Fp* | 88620→89057 | L | 145 | *C. pomonella* granulovirus *fp* (AAK70778) (56% over 146 aa) |
| 107 | *Ligase* | 89054←90793 |  | 579 | *A. segetum* granulovirus ORF 110 (AAS82628) (49% over 565 aa) |
| 108 | ORF108 | 90681→90884 |  | 68 | *X. c-nigrum* granulovirus ORF 143 (AAF05257) (42% over 68 aa) |
| 109 | *bro-4* | 91049→92464 | E/L | 471 | *S. litura* nucleopolyhedrovirus hypothetical protein (AAL01807) (93% over 478 aa) |
| 110 | ORF 110 | 92697→94076 | L | 459 | *S. litura* nucleopolyhedrovirus unknown (AAL01808) (97% over 459 aa) |
| 111 | ORF 111 | 94116→96131 | E | 671 | *X. c-nigrum* granulovirus ORF 64 (AAF05178) (54% over 694 aa) |
| 112 | *bro-5* | 96336→97106 | E | 256 | *X. c-nigrum* granulovirus ORF 130 (AAF05244) (50% over 234 aa) |
| 113 | *bro-6* | 97187→98644 | E | 485 | *S. litura* nucleopolyhedrovirus *hypothetical protein* (AAL01807) (64% over 489 aa) |
| 114 | *fgf-2* | 98679←99875 | E | 398 | *X. c-nigrum* granulovirus ORF 144 (AAF052258) (37% over 374 aa) |
| 115 | *alk-exo* | 99937→101163 | L | 408 | *A. segetum* granulovirus ORF 115 (AAS82623) (47% over 380 aa) |
| 116 | *hel-2* | 101165→102478 | L | 437 | *X. c-nigrum* granulovirus ORF 146 (AAF05260) (56% over 444 aa) |
| 117 | ORF 117 | 102448←103401 |  | 317 | *A. segetum* granulovirus ORF 117 (AAS82621) (33% over 295 aa) |
| 118 | ORF 118 | 103502→104482 | L | 326 | *X. c-nigrum* granulovirus ORF 147 (AAF05261) (44% over 238 aa) |
| 119 | *lef-8* | 104475←107024 | E | 849 | *X. c-nigrum* granulovirus ORF 148 (AAF05262) (68% over 859 aa) |
| 120 | ORF 120 | 107096→107458 | L | 120 | *X. c-nigrum* granulovirus ORF 165 (AAF05279) (44% over 109 aa) |
| 121 | ORF 121 | 107445→108620 | E | 391 | *38 kDa* (YP_762426.1) (31% over 295 aa) |
| 122 | *cg30-2* | 108776→109858 | E | 360 | *Mamestra. configurata* nucleopolyhedrovirus *cg30* (AAM09208) (23% over 216 aa) |
| 123 | *odv-e66* | 109897→112044 | E/L | 715 | *Hyphantria cunea* nucleopolyhedrovirus *odv-e66* (ABE73295) (23% over 643 aa) |
| 124 | ORF 124 | 117629←117838 | L | 69 | *Autographa californica* nucleopolyhedrovirus ORF 111 (AAA66741) (53% over 66 aa) |
| 125 | ORF 125 | 117999→118496 | L | 165 | *C. pomonella* granulovirus ORF 134 (AAK70794) (42% over 132 aa) |

Table S1. Continued

| ORFs | Name | Position | Promoter  motifs | Length  (aa) | Best-matched baculovirus ORF (Protein ID) (% aa identity) |
| --- | --- | --- | --- | --- | --- |
| 126 | ORF 126 | 118476←119426 | E/L | 316 | *X. c-nigrum* granulovirus ORF 172 (AAF05286) (34% over 316 aa) |
| 127 | ORF 127 | 119434←119661 | E/L | 75 | *A. segetum* granulovirus ORF 125 (AAS82613) (43% over 57 aa) |
| 128 | *lef-10* | 119615→119851 | E/L | 79 | *C. pomonella* granulovirus ORF 137 (AAK70797) (43% over 64 aa) |
| 129 | *vp1054* | 119700→120662 | L | 320 | *X. c-nigrum* granulovirus ORF 175 (AAF05289) (52% over 316 aa) |
| 130 | ORF 130 | 120710→120892 | E | 60 | *X. c-nigrum* granulovirus ORF 176 (AAF05290) (54% over 61 aa) |
| 131 | *fgf-3* | 121098→122036 |  |  | *X. c-nigrum* granulovirus ORF 178 (AAF05292) (38% over 297 aa) |
| 132 | *me53* | 122090→122959 | E | 289 | *P. xylostella* granulovirus ORF 120 (AAG27418) (40% over 304 aa) |
| 133 | ORF 133 | 123012→123317 | E/L | 101 | *Transcriptional regulator, MarR family* (ZP_00741471.1) (28% over 70 aa) |
