# Supplementary material for: Genomic Sequence Analysis of Granulovirus Isolated from the Tobacco Cutworm, Spodoptera litura
Source: PLoS One. 2011 Nov 23;6(11):e28163. doi: 10.1371/journal.pone.0028163 (PMC3223241; doi:10.1371/journal.pone.0028163)
Supplement: Table S2 — Primers used to confirm new SpliGV genes. (DOC) [file pone.0028163.s002.doc]

Table S2. Primers used to confirm new SpliGV genes

| Amplified target | Primer name | Primer sequence |
| --- | --- | --- |
| Spli30 | Spli30F | 5'- ATGAAGCAGCAGTTGATAC - 3' |
|  | Spli30R | 3'-TTATCGTAAACTTCTTGAGG- 5' |
| Spli40 | Spli40F | 5'-ATGGAACACTTGTCGAG- 3' |
|  | Spli40R | 3'-TTATTTAACCTTTTTCATTTCG- 5' |
| Spli51 | Spli51F | 5'-ATGGCGCATCATAAGTA- 3' |
|  | Spli51R | 3'-CTAAGTACCATAAGTCATGAGC- 5' |
| Spli61 | Spli61F | 5'-ATGGTTTACAGTTACTTGTGC- 3' |
|  | Spli61R | 3'-TTAATCATCATAACTATCAATCAAC- 5' |
| Spli63 | Spli63F | 5'-ATGGCTAAACGCAACAA- 3' |
|  | Spli63R | 3'-TTACAATTTTTGCACGTATACA- 5' |
| Spli75 | Spli75F | 5'-ATGGAGCACAAGTTTATCAG- 3' |
|  | Spli75R | 3'-CTAGATAAAAAAACTATTATTATCTTCATA- 5' |
| Spli88 | Spli88F | 5'-ATGTACATTTTAAAGTTGAACAAG- 3' |
|  | Spli88R | 3'-TCAATACACAAGCTCGAC- 5' |
| Spli121 | Spli121F | 5'-ATGGATCATATTGACTTTAGC- 3' |
|  | Spli121R | 3'-TCATAGTAGTAGAAATATAATTGCTG- 5' |
| Spli133 | Spli133F | 5'-ATGAAGCAGCAGTTGATAC- 3' |
|  | Spli133R | 3'-TTATCGTAAACTTCTTGAGG- 5' |
